# Sexual risk and HIV prevention choices among men who are mobile for work: Results from a rapid ethnographic assessment for the Mobile Men trial in South Africa and Uganda

**DOI:** 10.1371/journal.pgph.0005213

**Published:** 2025-09-18

**Authors:** Thabang Manyaapelo, Martin Mbonye, Seluleko E. Ngcobo, Dumsani Gumede, Julie Fox, Janet Seeley, the Mobile Men Study Team

**Affiliations:** 1 Social Science Unit, Africa Health Research Institute, KwaZulu-Natal, South Africa; 2 Social Science Programme, MRC/UVRI and LSHTM Uganda Research Unit, Entebbe, Uganda; 3 Desmond Tutu Health Foundation, based at the University of Cape Town, Cape Town, South Africa; 4 Department of Infectious Diseases, King’s College London, London, United Kingdom; 5 Global Health and Development Department, London School of Hygiene & Tropical Medicine, London, United Kingdom; Worcester Polytechnic Institute, UNITED STATES OF AMERICA

## Abstract

In preparation for a trial implementing oral and long-acting Pre-Exposure Prophylaxis [LA-PrEP] for mobile men in South Africa and Uganda, we conducted a rapid ethnographic assessment at each trial site between November 2023 and July 2024. We present findings from that assessment on men’s views on sexual risk and HIV prevention options which informed the trial. Each rapid ethnographic assessment took 15 days of continuous work in each community using a set of participatory methods: transect/spiral walks, ethnographic observations, conversations, in-depth interviews (IDIs) and focus/natural group discussions (GDs) with community members, health service providers and mobile men. We conducted 50 GDs, 61 IDIs and 297 informal conversations with men and women from four sites in Uganda and five in South Africa. Mobile men at the study sites were engaged in the transport industry (as taxi and truck drivers), construction and fishing. Many of these men were reported to be engaged in multiple sexual relationships. Being away from home, boredom and consumption of alcohol were reasons given for having many partners. Sex without a condom was widespread and was the preferred choice of the men because, they said, of increased sexual pleasure. None of the men was using PrEP. Men often said that PrEP was just for women. When given information on injectable and oral PrEP many men said that they would prefer injectable PrEP to avoid having to carry pills whilst travelling and, in addition, they would not have to remember to take pills. PrEP offers a convenient HIV prevention option for men who are mobile for work. The desire for condomless sex and the availability of oral and injectable PrEP may increase uptake. However, the associated risk of other sexually transmitted infections and blood borne viruses linked to not using a condom requires preventive interventions.

## Introduction

Men have consistently fared worse than women in levels of HIV testing, ART initiation and adherence [1,2] with significant challenges for engagement and retention in HIV prevention and treatment programmes in sub-Saharan Africa [3–5]. Men who are mobile for work in sub-Saharan Africa, a large diverse group made up of truck and taxi drivers, casual labourers, fisherfolk among others [6–11] are at particular risk of acquiring HIV [12–17].

While some groups of men who are mobile for work, such as truckers, have been the focus of research on the HIV epidemic in Africa [15,18], most HIV prevention research in Africa has focused on adolescent girls and young women [1,19]. There are a limited number of tailored HIV-prevention and care interventions for men who are mobile for work [20,21], but many of these men remain unreached [1,5]. Accessing HIV services regularly to receive prevention and care are a particular challenge for mobile populations [22,23], not only because of work schedules but also because of concerns about visiting clinics and carrying pre-exposure prophylaxis (PrEP) or anti-retroviral therapy with them as they travel [24–26]. Without men being able to access and take up HIV prevention methods, the elimination of HIV at a population level will not be attained [27]. Long-acting injectable cabotegravir (LA-PrEP) and on-demand oral PrEP offer HIV prevention methods which could meet the need of men who are mobile for work. These methods have not yet been evaluated in men living in high-burden settings who mainly have sex with women.

A rapid ethnographic assessment was undertaken in the context of a planned implementation trial. The oral (on-demand and daily) and long-acting Pre-Exposure Prophylaxis in mobile men in sub-Saharan Africa (Mobile Men) study is a mixed method, multi-setting, open-label randomized controlled trial with 400 HIV-negative men aged 18 years and above in South Africa and Uganda. Men will be randomized into two groups: Group A will receive oral tenofovir disoproxil/emtricitabine (TDF-FTC) PrEP (using either on-demand or daily) and Group B will receive long-acting injectable cabotegravir (LA-PrEP) over nine months. Following this, participants from both groups will be offered a choice to switch to their preferred method of PrEP for the last nine months of the study.

The rapid ethnographic assessment was done before the trial began to document the daily living and work arrangements of mobile men in the study areas and assess the existing HIV prevention and care options in the communities where prospective participants stayed. In this paper, we present the findings of the formative work conducted prior to the implementation of the trial. We report the men’s views on their access to HIV care and prevention services and examine men’s understanding of PrEP in relation to their perceptions of sexual risk, particularly in the context of new HIV prevention methods that offer protection against HIV infection. We also explore men’s views on LA-PrEP as a new HIV prevention method in their communities introduced through the Mobile Men study.

## Settings

The Mobile Men study is being conducted in two places in South Africa (Eastern Cape and KwaZulu-Natal provinces) and one place in Uganda (Masaka district). In each place we conducted the rapid ethnographic assessment in two to four sites, identified in this paper with the letters A-D. In the results section we preface the site letter with Ma, EC and KZN, standing for Masaka, Eastern Cape and KwaZulu-Natal, respectively.

We describe these sites in detail in S1 Text, with a brief overview here:

## Uganda

Site A: a fishing community in Masaka District on the shores of Lake Victoria. The population, most of whom are aged under 40 years, are engaged in fishing-related activities or working in bars, restaurants and shops serving the community.

Site B: a fishing community located about six kilometres from Site A. Similar to site A, but a ferry port serving the islands, so travellers often stay overnight and use lodges and bars/restaurants.

Site C: a transit town with travellers coming from western Uganda, and fishing sites, going to Kampala and beyond. There are several guest houses and bars/restaurants. Traders and pleasure seekers spend nights at the numerous guest houses which also attract large numbers of sex workers.

Site D: an island located about four hours from the mainland and close to the border with Tanzania. It is a popular location for fishermen during the fishing season. It serves as a transit route to other islands.

## South Africa

### Eastern Cape

Site A: a taxi terminal in the business area of a large city in the province. The place is busy because the taxis connect people using rail, road and the sea, coming to the area for work or commerce.

Site B: a taxi terminal in a township located 15 kms from Site A. The taxi rank has many private cars used as taxis – known as `cockroaches’ because there are so many carrying people in the local area.

Site C: is a construction site located about 32 kms from Site A. As well as a population living in informal and low cost housing there are also many temporary workers on the construction site, who move there for work.

### KwaZulu-Natal

Site A: this is a taxi terminal in the centre of a town in the north of the province. The town has a population of about 30,000 people. The site serves both local and long-distance travellers. Some of the taxi drivers are, therefore, from other parts of South Africa.

Site B: has three separate areas – a small town (with a population of about 3000 people), a timber yard on the border of the town and a service station/truck stop about 15 km from the town, used by long-distance travellers, including truck drivers.

## Study methods

### Ethics statement

The study was approved by the University of KwaZulu-Natal Biomedical Research Ethics Committee (BREC/00005745/2023), the London School Hygiene & Tropical Medicine Ethics Committee (LSHTM Ethics Reference: 29902), the University of Cape Town Ethics Committee (HREC Ref: 496/2023) the Uganda Virus Research Institute Research Ethics committee (GC/127/982) and the Uganda National Council for Science and Technology (HS3099ES). Written (for focus group discussions and individual interviews) and verbal (for informal individual conversations and natural group discussions) informed consent was taken from all participants before they engaged in an activity.

### Rapid ethnographic assessment method

The rapid ethnographic assessment is an approach using a mix of qualitative methods in a set sequence, designed to move from a broader to a more specific understanding of the topic being addressed. Through this approach we collected qualitative data that is both focused on the whole community context and also specifically on men and their HIV-prevention choices. The methods are described in more detail in S2 Text. The first rapid ethnographic assessment took place from 28^th^ November 2023, and the last one was concluded by 30^th^ July 2024.

The rapid ethnographic assessment methodology comprises of a set of structured activities presented in Table 1 below, aimed at gathering a comprehensive perspective on the area of study and the communities living there, in response to key research questions about the settings. The rapid ethnographic assessment approach we used is conducted in a limited timeframe of 15 days [28] by a team of six to eight research assistants, fluent in the local languages and trained in the data collection methods. This data collection methodology was standardized across the study areas to include observations, spiral or transect walks, individual conversations, in-depth interviews (IDIs) and natural group discussions (NGDs) and focus group discussions (FGDs) [29,30]. To facilitate community entry the research teams in the respective places engaged the Community Advisory Boards overseeing the chosen research sites for guidance on appropriate community entry specific to the sites concerned [31,32]. This was followed by seeking the relevant permissions from the local leadership, including traditional, political or community leaders depending on the area concerned [33], before data collection commenced.

**Table 1:** Research activities, key questions and methods used.

Research Activity	Key research questions	Methods
Community entry	What is the leadership structure of the site? How are the people organized? Who should we meet?	Through the guidance given by the local leaders/community advisory board members, initial introductory meetings are held with community leadership. Subsequent meetings are led by community leaders to introduce research teams to relevant community structures and fora.
Transect/Spiral Walks	What are the activities and movements (short-distance travel, commuting and longer migration patterns) of different age and gender groups? Where are the places of significance to HIV prevention in the community? PrEP knowledge and usage.	A transect/spiral walk starts from a central point, moving in concentric circles around the community and taking care to stop, listen, look and chat on the way. GPS coordinates and observation notes are recorded during the walk guided by a simple checklist (developed during the training).
Observations	What is happening in this community linked to HIV: risk-taking; economic activity; mobility, HIV prevention, PrEP knowledge and usage.	Observations carried out by researchers at different times and places in the community (choice of location based on information collected during transect/spiral walks). Activity reports and checklists are used to record details.
Individual conversations and natural group discussion	What are the HIV prevention, treatment and care options & stakeholders in this place? What is the history of HIV prevention, and HIV stigma in this place? What are the mobility patterns of men who move around for work in this community?	Participants are purposefully selected by the research team while conducting observations and transect/spiral walks within the community. Conversations are held with individuals or in groups casually around the research questions.
Individual interview	What are the HIV prevention, treatment and care options & stakeholders in this place? What is the history of HIV prevention, and HIV stigma in this place? What are the mobility patterns of men who move around for work in this community?	Participants are selected by the research team during walks and observations within the community, as well as recommendations from local leaders. Interviews are held with individuals on the same day or arranged for a time convenient to the respondent.
Focus group discussion	What kind of community is this? What are the local boundaries in this place? What is happening in the community in terms of HIV prevention? Are people aware of PrEP and what HIV prevention methods are used	Participants describe their community using pictures/words and help to draw a map showing local boundaries, places of significance to different groups of people (meeting places, main residential areas, etc.) and that identifies specific HIV prevention methods. Participants describe different types of people in the community and explore through discussion the possible influences on risky sexual behaviours.

### Data analysis

Data analysis was done manually using a framework analysis approach [34]. The aim and objectives which informed the design of the rapid ethnographic assessment were used to define initial thematic areas, with additional themes added if new ideas were identified from the study findings. These themes were shared and discussed across the sites to arrive at a shared coding framework. Following the identification of themes, indexing (coding) and charting (copying and pasting data according to thematic areas) were done simultaneously at each site. Mapping (visual display of data) was then done to allow researchers to identify patterns, associations, and concepts. Descriptive and analytical memos could then be produced from these charts. Each of the study teams in the three sites conducted regular staff debriefing sessions to synthesize and contextualise site specific findings. These meetings allowed for robust discussions at each site. In addition, weekly meetings by the social science leads (TM, SN, MM, JS) ensured that similarities and differences across the three sites were discussed.

Thematic content analysis was used to analyse and manage the interpretation of the data – working across all the sites using a shared coding framework. All interview and discussion transcripts and field notes, diagrams and maps were included for analysis. Each team produced an overview report of the findings from each site, for use by clinic team members prior to the commencement of the trial. For this paper the teams focused on the coded data on men’s sexual behaviour and their HIV prevention choices.

### Findings

The data for this paper are drawn from 50 group discussions (27 FGDs and 23 NGDs), 61 in-depth interviews and 297 informal conversations with men and women, as well as observational notes from the sites in South Africa and Uganda.

### Sexual behaviour and HIV risk perceptions

Being away from home, boredom and consuming alcohol were some of the reasons men gave for engaging in sex with casual, and sometimes, multiple partners. In the taxi ranks we found that some of the women from whom men got their evening meal also provided them with sex. *“We cook for them, and we give them sex”* (EC-Site B, FGD). At the fishing sites some of the young women serving in bars and restaurants made an income from providing sex to customers, with bar owners exploiting this by paying low wages to the women and keeping staff for limited time periods because `new’ women would attract customers. These women in both Uganda and South Africa would not necessarily consider themselves sex workers. There were other women in each site who provided sexual services as their main occupation often operating from particular areas in each site. One of the taxi drivers in KwaZulu-Natal explained the behaviour of men like him:

*“You find that we are sitting and bored and drinking alcohol, ooh yes we do drink, after drinking alcohol we get aroused, I then move to a place where I can find someone to have a nice time with, not that anyone is forcing you of course, we are old enough to do things because we want to do them”* (KZN, Site A, IDI).

A construction worker in the Eastern Cape gave a similar explanation:

*“Girls from the bar, we do not use condoms because these things just happen abruptly as we are drunk at the time you just take her to your place, it is the first time meeting her, she is from around the place, so you do not know her fully, the way she carries herself, her behaviour. You simply take her home and sleep with her and obviously without using a condom, so that is how these things happen, you are then at risk of contracting HIV/AIDS.”* (EC-Site C, IDI).

Nearly all the men we spoke to preferred sex without a condom because of increased sexual pleasure, which they considered a condom interfered with. One man from Eastern Cape explained that *`the plastic can waste the excitement’* (EC-Site C, FGD)*.* When asked about condom use, another man from Eastern Cape stated,

“*Most men do not; men have this saying ‘ayityiwa sweets ezine phepha’ (you do not eat a sweet with its paper on). Therefore, they cannot use a condom because they do not enjoy sex.”* (EC Site C, IDI).

Men in all sites used similar language to explain why they did not like condoms. The general sentiment pointed towards a shared recklessness for inconsistent condom use and multiple sexual partnerships. This is further illustrated from a quote from a man participating in a focus group discussion in Masaka who used the same example of not eating a sweet in its wrapping, as the one from Eastern Cape, above:

*“you can get a partner and choose to be with her, you know the youth have a motto that `sinunira sweet mu kaveera’ implying that they never want to use condoms to maximize sexual pleasure.”* (Masaka Site B, FGD).

Interestingly, the reputation of men for taking risks, something respondents in Uganda said was associated with men in fishing communities, seemed to fuel high-risk sexual behaviour, as if they had a reputation to maintain. A few men in South Africa rationalised this behaviour as keeping their sexual appetite high for when they were with their wives. They described this behaviour as looking after their `axe’ so it can be used effectively when at home. A man from Eastern Cape explained: `*If you don’t sharpen your axe, it stops cutting, it will go blunt, you will be with your wife at home day and night and your axe won’t work, it won’t come up (erection).’* (EC-Site B, FGD).

Some men did talk about condom use, indeed the same man who talked about the condom spoiling the excitement, mentioned above, said that he did sometimes use condoms, explaining how he made that decision:

*“You know as men we gauge women. Sometimes when having sex and the girl asks about a condom you would lie and say that you don’t have it, knowing very well that you have it in your pocket. And [then with] some women you would rather go knock at your neighbours and ask for a condom. That is how it is. You don’t just risk and go without it with some women.”* (EC-Site C, FGD).

He went on to observe that this method is not always reliable because one could not be sure a woman was not living with HIV.

The women who sold sex commercially at each site also made choices about the risks they took with the men, although when money was short, they may be forced to accept condomless sex in order to earn a higher rate. However, many of these women were familiar with oral PrEP having been targeted by HIV prevention organisations and were using it to protect themselves from HIV acquisition, but understood that condomless sex exposed them to the risk of other sexually transmitted infections. A female sex worker from Masaka explained the complexity of using condoms, suggesting that PrEP offered a better protection option:

*”We have to protect ourselves against HIV infections and I think a person has to take PrEP to protect against the HIV infections […] When a person comes and wants unprotected sex you can’t insist on protected sex. We use condoms but sometimes the men pierce them and, in the end, semen enters your body. If you are on PrEP you are safe. Condoms protect us against infections such as syphilis and candidiasis. Even if PrEP is available if a person doesn’t want to take then you can’t force but those who love their lives will take them.”* (Ma-Site A, FGD).

Another woman at the same site illustrated the challenges of negotiating condom use with men who were reluctant to use them:

“*… a circumcised man […] may come and insist on live (condomless) sex because he is circumcised claiming he is safe, yet he is infecting you. I still insist that let us stick to two things, condoms and PrEP. Do not listen to someone if his protection defence is because he is circumcised.”* (Ma-Site B, FGD).

These women were conscientious about their own sexual health; they understood the risks that their livelihood posed. Sex workers in South Africa were said to always have condoms on them One man from KwaZulu-Natal noted:

*“We do buy sex but we do not make a mistake, they always carry their condoms (sex workers),it is always up to a person if you decide not to use a condom especially if you know that you are already infected with HIV and you do not care. But if you know that you do not have HIV you always use a condom, as for me I always make sure of that***.***”* (KZN-Site A, IDI).

Relying on women to manage HIV-prevention may be linked to a sense that women had more ready access to prevention methods and healthcare in general.

### Access to healthcare

Men in all the sites explained that they associated health clinics with women and children. Taxi drivers in Eastern Cape, for example, complained that clinics: `*prioritise new mothers and those who come for family planning’.* When asked about the services they are aware of in their area, the men in KwaZulu-Natal said that where health care services were offered, they did not feel as if those services were intended for them.

*“Yes, there are people who came here. I do not remember the month but it was on women-based campaigns. It is unusual here at the rank to find a place that offers some assistance (for men).” (KZN, Site A, IDI).*

The men also explained how distance and a lack of time impeded access:

*“We do have a public clinic here in town, but it’s very far, and it’s always packed, you can queue in the line there for 4 to 5 hours. There is no care in that clinic.”* (EC-Site A, IDI).

“…*What I’m trying to say is that we are defaulting because health [centres] are far from us. […] that brother is saying someone once died here. Another person was driving and parked and died inside his taxi. […] he died sitting in his taxi. Another person was driving, he parked and said, “I’m not feeling well”. That was the last time we heard from him. The car was parked in the road*” (EC-Site B, FGD).

There were similar concerns among the taxi drivers in KwaZulu-Natal, with men attributing their lack of healthcare access to their busy work schedule:

*“Taxi drivers do not have time. You see taxis do not stay here (at the terminal) for very long, you find that if you try to go say to the mobile clinic (on the outside of the town), as soon as you get there you can get called back because your taxi is now full and you need to deliver the passengers. When you get to the destination, you are called back again to the terminal.”* (KZN, Site A, IDI)

Fishermen in Uganda also explained how clinic location, opening times and queues impeded their health care access, including their access to health care information. From a group discussion in MA, Site B, one participant echoed the sentiments about women being more likely to access services: *`The people who test more are the women, and the reason is that by the time those services come to the area, they [women] are not busy.’* (MA, Site B, FGD)*.*

This view was shared by a health care worker in another group discussion in MA, Site B: *`I see that women take responsibility for themselves and their children to seek medical help early as compared to men’.* (MA, Site B, FGD).

### Knowledge of PrEP and HIV prevention choices

None of the men in any of the sites that we spoke to was using PrEP at the time of the study. Some knew of oral PrEP but thought it was for women (to protect a child in pregnancy). As an introduction to the rapid assessment, the study team shared information on different types of PrEP, as well as the types of care available for people living with HIV. We found that PrEP (used by women) was often confused with anti-retroviral therapy or post-exposure prophylaxis. When told about oral PrEP, one man from Eastern Cape observed:

*“No this is news to me, I only heard about it from pregnant woman that they should take the pill so that they do not transfer the virus to the unborn baby, but this is nice to hear [that it is] also even for men, because men do not trust themselves.”* (EC-Site A, IDI).

The reason men gave for not using oral PrEP was because of a lack of information, largely because, as noted above, they did not go to health centres or clinics.

*“…I usually hear about PrEP from females, saying they are going to the clinic to get PrEP. So, I did not know that men are also allowed to take PrEP.” (EC Site C, FGD).*

Consequently, due to the lack of knowledge and misinformation about PrEP, there was some confusion between types of HIV prevention and treatment (because both anti-retroviral and PrEP pills were seen as being the same). A local health worker at Site A in Masaka, talking about the fishermen, said that:

*“They have a perception that PrEP is a virus that when you take it you get infected and that the drug was manufactured with a virus and that the moment you take it you get infected [...]. Those are the perceptions they have that they [unspecified] want to reduce the people so that they die in plenty.”* (Ma-Site A, IDI).

The main concern the men had was that if they were seen taking pills, then it would be assumed that they were living with HIV. The idea of a long-acting injection that provided protection from HIV was welcomed by many of the respondents when they were told about it:

*“PrEP tablets may lead to stigma, and you can end up losing your friends as they may be tempted to think that you are HIV positive. But with injectable no one will ever know about your presence on injectable PrEP. It’s always between you and the health worker.”* (Ma-Site A, FGD).

A local leader in Masaka commented

*“Based on myself, I can’t go for tablets when I have an alternative of injection […] If the injection is not daily, and if I can get it at least for three months and am sure that it’s preventive, I rather get an injection but not tablets. And even in normal sickness people prefer using injections over tablets.*” (Ma-Site B, IDI).

None of the men we spoke to in any of the sites had heard of injectable PrEP before the introduction of the study. However, health workers at all the sites mentioned that people, although not necessarily the mobile men, had heard about an injection in the media and were asking when it would be available. There were some concerns. For example men from Site B in KwaZulu-Natal as well as from Site A in Eastern Cape voiced reservations about LA-PrEP because of the fear of injections and the distaste they felt over someone touching their bottom to administer the dose. However, they thought it would be a good option for men like them who travelled and could not carry pills with them, because the pill bottles rattled, revealing to those around them that they were carrying some form of medication, which they assumed people would associate with HIV treatment.

In the taxi rank in Eastern Cape several men said that injectable PrEP could become the preferred method of preventing HIV because men were not going to change their sexual behaviour. There was general optimism that this form of PrEP would be a game changer for men in general and for those who were mobile for work because they were used to engaging in risky sex. However, there was some misinformation about the benefits of taking PrEP:

*“…It is said that this PrEP makes you sexually active, you always need a woman when you take PrEP, that is the reason we are interested in hearing more about it because we know….as men, we know when we talk of PrEP…laughter…I’m sure I’m not the only one who knows about this.”* (EC Site A, FGD).

In summary, mobile men in our study did not tend to access health care facilities because they perceived them as unwelcoming to males. They did not like the long queues because the nature of the men’s work did not afford them time to wait to access health care. These men often engaged in sex with casual partners, without using any form of HIV protection. A dislike of condoms because of the effect on their sexual pleasure and a wariness about taking pills for prevention because of the association with HIV treatment, as well as being viewed as a method for women to use, led to a general reluctance from men to take up these prevention methods. However, the idea of a long-acting injection, which meant that they would not have to take pills and could have condomless sex, was viewed with enthusiasm because of the promise that the method held to provide protection from HIV without having to change their sexual behaviour.

## Discussion

In this paper we provide insights into the sexual risks taken and faced by men who travel for work and how injectable PrEP may fit into HIV prevention for these men. Our findings corroborate the results from other studies looking at people’s willingness to accept injectable PrEP, including for men [35–37]. As Cheng and colleagues [35] observe, facilitating the adoption of a new form of protection requires understanding what the potential client group need. The men in our study, in all the sites voiced their concerns about other forms of protection. Such concerns particularly about condoms have been reported elsewhere [38–40], highlighting the need for other HIV and other sexually transmitted infection prevention options. Other concerns the men raised speak to the barriers they perceived in accessing health care such as clinics prioritizing female reproductive health and being too far away, corroborating the findings of previous studies [41]. A recommendation to the implementation trial team from the rapid ethnographic assessment findings was to bring services closer to where the mobile men worked to provide better access [42].

The ways in which men rationalise the risks they take in their sexual behaviour, including being reluctant to use methods associated with HIV prevention and treatment, are associated with a need to demonstrate being a `real’ man, this can be particularly the case if they feel unable to get a job, or to be able to earn adequate money to support a family or girlfriend [43–45]. This form of manhood has been termed reputational masculinity as men seek the approval of their peers, and the women they want to attract, by demonstrating sexual prowess and the absence of fear of illness [46,47]. Boredom and social consumption of alcohol together with other male companions were cited by the men as their reasons for engaging is risky sexual behaviours. Alcohol has been shown to increase risk taking behaviour [48] and reduces the ability to use condoms. Whilst boredom may not be associated with risk taking, it still contributes to poor choices about HIV protection and sex [49]. As some of the female sex workers we spoke to noted, even if these men do use PrEP, they still expose themselves and the women they sleep with to other sexually transmitted infections if they do not use a condom [50]. The condom usage by the sex workers we spoke to was likely to have been exaggerated [51,52] due to social desirability bias. However, condom use is a complex subject especially for female sex workers who must constantly negotiate between paying clients and non-paying partners with whom consistent condom usage has been shown to be lower [53].

However, the introduction of injectable PrEP as a method of protection which men, such as those in our study, may use, is an important step towards meeting the goal of ending AIDS by 2030 [54], protecting both the men who use it, and their casual and long-term partners from HIV acquisition.

Our findings highlight the concern that while PrEP is perceived to be highly effective as a form of protection against HIV acquisition, the method does not safeguard against other sexually transmitted infection and unplanned pregnancies [55–57]. Our findings suggest that while LA-PrEP may offer a practical way for men who are mobile for work to access HIV-prevention, there is a need for awareness raising on risks from other sexually transmitted and blood borne infections, for which using a condom can provide protection. That said the message that using PrEP, in any form, does not replace condoms but adds to sexual health protection methods, will be difficult to convey to men in settings where condomless-sex is seen as desirable to enhance a man’s pleasure.

### Strengths and limitations

A strength of our study is the large number of men we were able to gather detailed information from on HIV prevention. However, these men were by definition mobile and were not always available for follow up conversations. The time spent talking to some men was often very limited because they were in hurry and that may have influenced some of the responses. However, engaging with natural groups of men in their place of work or leisure enabled us to adapt our approach to men’s lifestyles and schedules for data collection. The views and perception of members of these communities who were less interested in participating may have been underrepresented despite efforts to select across all sectors of the community. Lastly, more time in the communities at the respective sites, beyond the 15 days would have allowed for a larger number of conversations about perceptions on PrEP and HIV prevention, however the approach is thorough and designed to gain an overview of a setting prior to the commencement of a trial, and further social science research (which is embedded in the trial) [30].

## Conclusion

Men who are mobile for work may have few choices about the type of work they can do, in places with high unemployment as in both Uganda and South Africa. They do, however, make choices about their sexual behaviour in the places they stay, sometimes choosing condomless sex because of assumptions about the type of woman they have chosen, and also because of the additional pleasure sex without a condom gives them. As a prevention choice, injectable PrEP holds considerable promise for men who are mobile for work, particularly as there was a reluctance to take oral PrEP. However, it is yet to be seen whether men who are mobile for work can attend on time for their follow-up injections; LA-PrEP may provide freedom from pills, but clinic attendance is still required.

## Supporting information

S1 Text Study settings. (DOCX)

S2 Text Methods. (DOCX)
