# Supplementary material for: Sexual risk and HIV prevention choices among men who are mobile for work: Results from a rapid ethnographic assessment for the Mobile Men trial in South Africa and Uganda
Source: PLOS Glob Public Health. 2025 Sep 18;5(9):e0005213. doi: 10.1371/journal.pgph.0005213 (PMC12445478; doi:10.1371/journal.pgph.0005213)
Supplement: S1 Text — (DOCX) [file pgph.0005213.s001.docx]

**S1 Text – Study settings**

The study was conducted in three sites (one in Uganda and two in South Africa). The Ugandan sites consisted of three fishing communities and one in-land town located along a major highway connecting Uganda to countries such as Rwanda, the Democratic Republic of Congo and Tanzania. One of these sites was an island community. The South African sites were in two different provinces: Eastern Cape and KwaZulu-Natal. The Eastern Cape Province sites were a taxi rank in a port city, a taxi rank in a township located about 15 km from the city and a low-income housing development construction site. The KwaZulu-Natal province sites were a taxi rank in a rural town and a small town with a truck stop and a timber yard along a main highway.

**Uganda**

**Site A** is a landing site located just north of Lake Victoria. It is made up of five communities that are governed by a local council. The population varies depending on the fishing season but at the time of the rapid assessment (non-fishing season) it was estimated at about 3,000 people. Most of the population are adult men and women engaged in fishing activities. The land size of this community has been shrinking due to the rising water levels of the lake and there were some housing structures that were observed to be submerged by the water. The area is served by one main private health facility that is used by both the Government and Non-Governmental Organisations (NGOs) to channel health services to the population. Within the community, there are smaller drug retail shops. HIV care services, including anti-retroviral therapy, can be accessed at health facilities. Female sex workers who work in the area are attracted to the site because they are assured of earnings, especially during the fishing season. Populations from nearby fish landing sites and islands frequently come to this site for trade or entertainment. There are many bars and lodges at the site. Most houses in this community are temporary and the main construction material used for building is iron sheets, used for both the walls and the roof, making them quite hot during the dry season.

**Site B** is a smaller landing site about 6 km from Site A. This site, too, is shrinking due to recent (since 2019) flooding that has led to the relocation of many from the community to a roadside town about 2 km away. This community is served by a government health facility that is better staffed than the facility at Site A. The population of this community is estimated at around 1,000 people and it also fluctuates depending on the fishing season. There is a busy main road that leads to a ferry, making this a transit town for many people who regularly rely on the ferry to move to and from nearby islands. These travellers make use of bars, restaurants and lodges as they wait for the ferry or on arrival from the islands.

**Site C** is an island community located in Lake Victoria close to the border with Tanzania. The distance is often calculated by local people in terms of time required to reach the island. It took the research team nearly 4 hours to reach this place from the mainland. The island has a small population of about 500, which increases during the fishing season. There is a small population who call the island home; most people travel to the island for short visits en route to and from other islands with fewer facilities. There is a small health facility offering services supported by government and NGOs.

**Site D** is a town with a population of about 40,000 people located on the edge of Masaka District. It is a busy town that serves as a stopping place for long-distance truck drivers, a hub of large- and small-scale businesses, and a stopover town for buses moving to and from different parts of the country. Fish products from the fishing community are brought to this community for sale. There are many restaurants and bars in the town. Lodges and guest houses offer cheap accommodation and some provide sex work venues.

**South Africa**

**Eastern Cape**

**Site A** is located in one of the largest cities in the area and serves as a major centre for commerce, industry, and services. There are multiple taxi ranks spread across the central business area in the city, offering residents and visitors access to residential areas and commercial spaces within and outside of the city. Site A is one particular taxi rank serving numerous routes with a sizable commuter population because of the nearby port and railway hub (for goods) as well as the road transport bringing goods and people to the area. The taxi rank is administered and managed by the local municipality governing the area.

**Site B** is also a taxi rank but located in a township about 15 km from Site A. The township is divided into low- and middle-class populations. The slow-growing township has new extensions made of informal houses (shacks) and formal houses through the support of the Reconstruction and Development Programme (RDPs), which is government-subsidized housing. There are also a number of self-built homes. Despite evidence of slow growth, the township is well-placed for access to the business area in the city. Much of the transport in the taxi rank is provided by “*amaphela*” (cockroaches). These cars, unlike minibuses, do not spend a lot of time parked as they only take 4-7 passengers. They live up to their cockroach name as they result in a high turnover of private cars used as taxis that cause traffic jams on the roads within the township.

**Site C** is a construction site for 1,500 RDP houses. These houses are often single-story, with basic amenities, and are designed to provide affordable shelter. The construction of the housing structure is formal, with bricks, concrete, and a tile roof. However, there are a few visible informal structures (shacks) located within the area that are currently being improved. We noted that the informal houses are congested, not properly built, and utilize illegal electric connections (*izinyoka*). We noted illegal powerlines connecting from the nearest electricity poles and extending to the informal settlement. The construction site workers are usually immigrants to the town, moving from construction site to construction site.

**KwaZulu-Natal**

**Site A** is a taxi rank located in a municipality situated in the southern corner of the province. The town has a population of about 32,000 people. The taxi rank has a main taxi rank and an older taxi rank. The main taxi area is divided into long-distance taxis and those doing local routes. Around the taxi rank are food outlets and general stores used by the travellers and taxi drivers. This main rank has armed guards on duty. The taxi rank is arranged along a 150-meter bend of the road going from the south to the north of the town. The southern wall is comprised of retail businesses and street vendors, and the northern wall is an open area with street vendors and one temporary health clinic. There is no visible security and structure of operations as compared to the main taxi rank. Drug dealing has been reported in this area. There is a large group of homeless young people who live in this area.

**Site B** is a town located about 50 km north of Site A. The site has a main town area with shops and residences and a population of about 4,000 people, a timber yard located at the edge of the town where lorries deposit and pick up loads destined for paper mills or export, and a service station/truck stop about 10 km from town, where long-distance truck drivers can rest and get food and drink. As a result of the timber yard and truck stop the town has a steady stream of truck drivers who use the facilities in the town and truck stop.
