# Supplementary material for: Sexual risk and HIV prevention choices among men who are mobile for work: Results from a rapid ethnographic assessment for the Mobile Men trial in South Africa and Uganda
Source: PLOS Glob Public Health. 2025 Sep 18;5(9):e0005213. doi: 10.1371/journal.pgph.0005213 (PMC12445478; doi:10.1371/journal.pgph.0005213)
Supplement: S2 Text — (DOCX) [file pgph.0005213.s002.docx]

**S2 Text – Detailed Methods**

Here we describe in more detail than in the body of the paper the methods and approach used during the rapid qualitative assessment in the different sites. A rapid qualitative assessment is conducted over a 15 day period and comprises of a set of structured research activities, aimed at gathering a comprehensive perspective on the area of study and the communities living there. The data collection and analysis is guided by four meta-indicators to provide an account of the places and the people who work and reside there. The meta-indicators included physical features, social organisation, networks and community narratives. Data were collected through observations, spiral or transect walks, individual conversations, in-depth interviews and natural and focus group discussions.

**Community entry**

In Eastern Cape, community entry was sought by engaging with a municipal body providing HIV support for men as a supportive mechanism for the Mobile Men study. The municipal body comprises community participants, men-focused community organizations, and municipal community outreach representatives. The team identified and agreed on areas to approach and locate the Mobile Men study through these consultations. This was followed by consultations with local councillors, and with their support, we approached taxi associations, rank managers, and construction site managers to introduce the study.

In KwaZulu-Natal, the study was presented to the Community Advisory Board of Africa Health Research Institute. This board is made up of members of the traditional and civil councils in the study district. Their advice was sought to determine the language and terminology acceptable to members of the community and how to build trust with the stakeholders. This was followed by an introduction of the study in the communities. This involved meetings to seek permission from the traditional leadership structures and municipal counsellors to work in the area. We then conducted community meetings and roadshows to explain the purpose of the study, giving members the opportunity to seek clarifications and get answers to any questions or concerns.

In Uganda community entry was sought by engaging area leadership through the existing structures. The area leaders identified the most relevant people who acted as the guides around the community because of their familiarity with the different categories of the population. The study was explained to the leadership who were then mobilized to participate in the community group discussion. Besides collecting data about the area from participants representing different sections of the communities, these discussions obtained community consent and offered advice to the research team about the area. This process helped the research team to build rapport with host community during the study period.

**Data collection approach**

The rapid assessment employed a set of qualitative research activities to quickly identify key community features and community perceptions around mobility and HIV risk to inform setting up of the Mobile Men Study. At each study site the data were collected by a team of research assistants over 15 days during the months of October 2023 to June 2024. Below, we describe the methods used.

The rapid assessment approach is centred on addressing pressing issues within communities [1]. This study focused on HIV vulnerabilities amongst mobile men, which has been neglected by research and interventions, with evidence showing heightened vulnerabilities to multiple sexual partnerships and HIV infections amongst men who move for work [2, 3]. These challenges for mobile men are deeply intertwined with residents' everyday experiences, making it necessary to address them holistically. This method of data collection offers diverse tools to capture the intertwined challenges and opportunities in communities and has been used successfully by studies elsewhere [4].

This methodology was particularly valuable as we aimed at creating social awareness for the forthcoming trial part of the Mobile Men Study on injectable pre-exposure prophylaxis (PrEP). Through this assessment, we informed the selection of communities for the trial.

*Community entry group discussions*

Introductory group discussions were held with area leaders and people who were knowledgeable about the topic of mobility and the history of the area. These discussions were often engaging and informed the research team about the areas of focus for the data collection. A map drawn by the participants during these group discussions laid the foundation upon which structured observations and spiral walks were built.

*Spiral (transect) walk*

A spiral walk uses a systematic approach moving from the centre (or as close to the centre as feasible) to the outskirts of the area under study, with the intention of getting an idea of the layout and conducting informal conversations with people met *en route*, to introduce the study. Immediately after the walk our team members wrote down the details of their walking route and documented their experience in field notes using an observation sheet. This method involved impromptu conversations, picture documentation, and GPS readings to pinpoint areas noted as important to the study [1]. The walks assisted the team in identifying potential interviewees and places to conduct structured observations. In all sites, the spiral walks were done in pairs by a male and female research assistant to encourage both men and women to chat to the research team as they walked around the area.

*Structured and Timed Observations*

When the spiral walk was completed, the researchers sought to understand the nature of the community activities. Timed observations were scheduled and conducted at different times of the day, categorized into morning, midday, and afternoon and at different times of the week, including the weekends. Night observations were also conducted. This was conducted systematically with explicitly formulated rules on what the research team should look for and how they should record it, using an observation schedule. In one of the sites, through observations conducted at different times of day we noted that taxi ranks providing short distance travel were busy in the morning and afternoon, making it easy to approach taxi drivers during the midday slot. While the long-distance taxis were busy from morning to midday, and some would return in the afternoon to rest. Thus, these drivers were best targeted after midday. These data were recorded on an observation sheet on which was noted not only the activity of the main actors in the area but also the people who came in and out of observed areas, noting their demographics, reasons for visits and other factors.

*Natural group and focus group discussions and informal conversations*

Natural group discussions and informal conversations were conducted when the teams identified potential interviewees during their observations and walks and by local leaders who suggested appropriate respondents. These sessions were either spontaneous or pre-arranged, some were more formal focus group discussions, where participants were invited to a particular location for the discussion. The nature of the group discussion (spontaneous/natural or focus) depended on the group of people being targeted and on the arrangements made by the study teams [1]. Taxi drivers, for example, who may be in a rush between jobs would be willing to join a spontaneous discussion for 15 to 20 minutes with a few other taxi drivers near their vehicles, but could accept an invitation to attend a focus group discussion. All the groups were guided by a semi-structured topic guide. The groups convened included men who were mobile for work, health care professionals as experts, and community leaders and local people, both men and women from varying age groups, with a particular focus on younger people (largely because of the age distribution in all the sites). During these discussions the teams probed for more information on some of the issues identified through spiral walks and observations, clarifying issues from the observations. We explored the type of community, dominant activities, availability and access to health and HIV-related services and the quality of life in the community and asked people to describe what type of place the community was in their own words.

*In-depth, key informant interviews*

These were interviews with people selected in consultation with community members, representing different types of people living in the place. These interviews were conducted to add to the description of the community through the perspective of mobile men and key stakeholders in the communities, such as healthcare professionals, community leaders and civil society. These interviews lasted between 60 -120 minutes, during which we sought to introduce the study and seek an understanding of the community, focusing on the awareness of the issues of the study such as health care access and uptake by mobile men and men’s experiences of HIV prevention.

**Reporting: Community Profile Report Writing**

Data collected during interviews and discussions were recorded with participants permission and transcribed within 72 hours of the data collection. Conversations and ad hoc natural group discussions, as well as observations and accounts from the spiral walk, were written up from field notes. The data were checked by the lead social scientist in each site. A report was then compiled from the data from each site. These reports were structured by four meta-indicators, following the guidance in Bond and colleagues [1]. Each site assessment was followed by a report of a multi-layered community description that included the following:

1. Physical features, which were concerned with the physical structure of the environment; through observations and spiral walks, we recorded the physical features of each site. We recorded the housing type and the transportation networks, such as roads, railways, and shipping (for Eastern Cape). In this component, we focused on the use of the site, noting the types of housing, whether business or residential use and supporting structures such as recreational spaces found within the sites. The topography of the area was noted, and the presence of water bodies and vegetation was recorded.
2. Social organisation which focuses on the people located in the site. We recorded the demographics of the population at the site, noting ethnicities, gender balance, attire, and activities that people were engaged in. These data were collected through observation, spiral walks, and during discussions and interviews.
3. Social networks are concerned with the interactions between people and between actors and the environment. We collected these data during discussions and interviews, learning how people were organised in the place and, in the case of men who were mobile for work, where they might get support from others when staying in the community under study.
4. Community narratives are the stories people tell about a place during conversations, discussions and interviews. Dominant narratives focused on issues such as masculine dominance and men’s preference for risky sexual behaviours and views on PrEP as a method of HIV prevention.

The writing up was completed in four days. It is from these reports that an evidence informed decision was taken to identify viable sites to situate the trial.

The table below illustrates the process that was followed across the sites in data collection.

**MOBILE MEN – OVERVIEW OF RAPID QUALITATIVE ASSESSMENT.**

| **Day** | **Research Activity** | **Aim/s** | **Key questions** | **Participants** | **Duration** | **Data Outputs** |
| --- | --- | --- | --- | --- | --- | --- |
| **0** | Pre-Fieldwork  Desk-based review and creating the base map.  Review of published and unpublished reports and papers, including looking at data from other studies which have been conducted in the study settings. | To gain a thorough understanding of some of the key characteristics of the community before fieldwork commences.  To enable researchers to decide on appropriate research community sites; while some studies might have communities pre-selected for research, other projects will require researchers to find out more before community sites are chosen. | What is known? | Local leaders and local organisations will be asked for advice/ideas on what sources exist in the search for documents on the study sites. | Not Specified. | A report of the base-level information in a word document; an excel spreadsheet containing a repository of contact details of all relevant organisations and stakeholders; and a base-map. |
| **1** | **Community representatives group discussion (natural and focus group discussions)**  Use the Community Representative Discussion guide. | Discussions with stakeholders/gatekeepers of relevance to the research topic  Aimed to gain entry to the community and assess key issues.  To use the community map as tool to plan the transect walk and discuss places of relevance to the research topic.  To elicit suggestions about where community observations should be conducted.  To ask for recommendations about key informants that should be interviewed. | What kind of community is this?  What is happening in the community in terms of livelihood options for men?  What health services exist? | 8-12 participants Community representatives.  Community advisory boards (CABs).  Community health committees.  Neighbourhood committees.  Community police forums. or other groups, (depending on the context and research aim). | 1-2 hours. | A map of the local community marked up with the route/s for the transect walk and places of particular interest to the research topic, a summary of the discussion and activities to feed into the community profile, scripts/transcripts for finer analysis. |
| **2-3** | **Spiral (transect) walk.**    Use Watch-Based Navigation system and Map to highlight areas and components noted. | To create a detailed description of the key physical features of a community and make observational notes of the movement of people, industries, economic activities, and a description of the demographic profile of the community. During the transect walk, researchers identify places where further observations can be conducted. | What are the activities and movement of different age and gender groups?  Where are the places of significance for men’s work and for HIV transmission and prevention in the community?  Where and how do men access HIV testing, HIV care, sexual health care, condoms, VMMC and PrEP? | Not specified | Approx:  2 days  -Morning  -Afternoon | Detailed notes on places of relevance, ‘breadcrumb trail, first impressions of the community for report writing, an updated community map.  Optional: GPS readings. |
| **4** | 1^st^ writing up day: Community profile and mapping report writing. | | | | | |
| **5-7** | **Observations**  Work sites, health services, significant events, weekend or evening gathering places, entry/ exit points | To note details of key places in the community, both in the general sense but also places specifically related to the research question. | What is happening in this community linked to: men’s work, wider economic activities, sexual risk-taking, mobility; HIV testing, care and prevention | Not specified | 30-60 Min | Detailed notes on places of relevance, completed observation sheets, and descriptions of the community for report writing.  *NB: Field notes should be written up immediately after the observation session as much as possible.* |
| **8** | 2^nd^ writing up day: Community profile and Mapping report writing. | | | | | |
| **9-11** | **Community group discussions group discussions (natural and focus group discussion)**   - **Specialist group** discussions (if necessary) - **Mobile Men** group discussions.   A semi-structured community group discussion guide is used to lead discussions. | To find out about community perceptions of the research questions/ issue.  To explore the groups’ perceptions of the research focus area in their community  To explore the group members’ direct experiences of the research focus area | What are community and individual perceptions of men’s mobility and the work they do.  What are their experiences with HIV prevention, treatment and care options? | 8-15 Participants  Age: 18-35   - Young men - Mixed - Community members (if necessary). | Approximately 1-2 hours per discussion | A summary of the discussion and activities to feed into the community profile, scripts/transcripts for finer analysis, and a list of relevant stakeholders. |
| **12** | 3^rd^ writing up day: Community profile and Mapping report writing.  Updated key information based on findings. | | | | | |
| **13-14** | **Key informant interviews**   - Semi-structured interviews. - Use Interview Schedule. | Semi-structured interviews with persons with experience and insight into the problem. | What kind of community is this?  What is happening in the community in terms of livelihood options for men?  What health services exist? | 4 Participants | 45-60 minutes per interview |  |
| **15** | 4^th^ Draft: Community profile and Mapping report writing.    Draft of site report ready within 4 (working) days | | | | | |

| **Eastern Cape demographics**  The age range of the men: ~20 – 65 years  Occupations: Long and short-distance taxi drivers, hawkers, Rank management, and Construction Workers.  15 in-depth interviews and 52 informal conversations with community representatives (men and women), long-distance taxi drivers, short-distance taxi (cab) drivers, and construction workers. ~15 (natural and focus group) discussions held with groups of these people.  **KwaZulu-Natal demographics**  The age range of the men: ~20 – 65 years  Occupations: Long and short-distance taxi drivers; truck drivers  15 in-depth interviews, 75 informal conversations with truck drivers, taxi drivers, cashiers, petrol attendants, firefighters, soccer players, street vendors, petrol station security, street vendors, taxi rank security, and taxi rank staff. ~15 (natural and focus group) discussions held with groups of these people.  **Masaka demographics**  The age range of the men:  ~18 – 45 years  Occupations: Fishermen, boat owners, traders, Long and short-distance taxi drivers; truck drivers, hawkers and commercial motorcyclists (boda-boda),  31 in-depth interviews, 170 informal conversations with people involved in fisheries and services in the fishing communities including women who sold sex. Shop workers, guest house and bar staff. ~20 (natural and focus group) discussions held with groups of these people. |
| --- |

**Data analysis**

Data analysis was done manually using a framework analysis approach. The aim and objectives which informed the design of the rapid ethnographic assessment were used to define initial thematic areas, with additional themes added if new ideas emerged from the study findings. These themes were shared and discussed across the sites to arrive at a shared coding framework. Following the identification of themes, indexing (coding) and charting (copying and pasting data according to thematic areas) was done simultaneously at each site. Mapping (visual display of data) was done to allow researchers to identify patterns, associations, and concepts, descriptive and analytical memos were produced from these charts.

Thematic content analysis was used to analyse and interpret the data – working across all the sites using the shared coding framework. All interview and discussion group transcripts and field notes, diagrams and maps were included for analysis. The analysis employed an iterative approach where the data were read several times to identify new themes which had not come from the topic guides. Meaning units were abstracted and coded, and the codes were compared for similarities and differences, a process that was revisited multiple times and then the codes were grouped into categories to identify the main themes. The final step included developing detailed analytical memos from the data for each theme.

For this paper we focused on the data on HIV prevention choices, and more broadly the choices men about the risks that they take in their sexual behaviour.

**References**

1. Bond V, Nel M, Simuyaba M, Chirwa T, Viljoen L, Chiti B, et al. The Broad-Brush Survey Approach. A set of methods for rapid qualitative community assessment. Zambia and UK: Zambart/LSHTM; 2023.

2. Cornell M, Majola M, Johnson LF, Dubula-Majola V. HIV services in sub-Saharan Africa: the greatest gap is men. The Lancet. 2021;397(10290):2130-2.

3. Medina-Marino A, Bezuidenhout D, Ngwepe P, Bezuidenhout C, Facente SN, Mabandla S, et al. Acceptability and feasibility of leveraging community-based HIV counselling and testing platforms for same-day oral PrEP initiation among adolescent girls and young women in Eastern Cape, South Africa. J Int AIDS Soc. 2022;25(7):e25968.

4. Ssemata AS, Ndekezi D, Kansiime C, Bakanoma R, Tanton C, Nelson KA, et al. Understanding the social and physical menstrual health environment of secondary schools in Uganda: A qualitative methods study. PLOS Global Public Health. 2023;3(11):e0002665.
